# Is misokinesia sensitivity explained by visual attentional orienting? ERP evidence from an emotional oddball task suggests no

**DOI:** 10.1371/journal.pone.0306464

**Published:** 2024-07-29

**Authors:** Sumeet M. Jaswal, Todd C. Handy

**Affiliations:** Department of Psychology, University of British Columbia, Vancouver, BC, Canada; UESTC: University of Electronic Science and Technology of China, CHINA

## Abstract

Misokinesia is a prevalent condition characterized by strong, negative emotional reactions to the sight of repetitive fidgeting movements in others. Here we present the results of a study designed to explore the relationship between misokinesia sensitivity (MKS) and attentional sensitivity to affectively-valenced visual stimuli. In particular, we asked participants with either high or low levels of MKS to perform an emotional oddball task that included responding to faces that had either angry or happy expressions, while we recorded event-related potentials. We found that there were no significant differences between the two MKS groups in attentional sensitivity to these faces, as indexed by the amplitude of the P300 ERP component they elicited. Importantly, we could not ascribe this null ERP finding to either low statistical power or the idiosyncrasies of our ERP analysis parameters. As such, our findings add to growing evidence that MKS may not be the simple result of heightened attentional orienting responses to visual events, but rather, it may be associated with other aspects of cognitive-affective processing.

## Introduction

Misokinesia is a psychological phenomenon defined by a strong negative affective or emotional response to the sight of someone else’s small and repetitive movements, such as fidgeting with a hand or foot [1]. Although approximately one-third of the general North American population appears to self-report at least some degree of misokinesia sensitivity (MKS), our recent reporting of this prevalence rate and data suggesting that misokinesia is not associated with altered attentional responses to sudden-onset visual events [2] remains the only published work to date specifically addressing the potential mechanistic basis for MKS. As such, we have yet to establish the cognitive and/or affective factors that may help to explain this widespread visuo-social sensitivity and inform intervention strategies. To address this lacuna, we thus performed the following exploratory study to assess the possibility that self-reported MKS may be associated with an overall heightened responsivity to affectively-valenced visuo-social stimuli.

Given the literal absence of empirical work on MKS, our study was informed by two lines of reasoning drawn from the literature on misophonia, misokinesia’s auditory counterpart which has received increasing research interest over the past decade [1]. First, although the strong negatively-valenced affective responses to auditory triggers in misophonia may modulate with the identity of the individual making the sound [e.g., a family member vs. a stranger; see 3–6] heightened affective reactivity itself is a core defining feature of the misophonic response [7–9]. Second, in a recent case study, Webb [10] reported that administering the *β*-blocker Propranol to an individual with severe misophonic and misokinesic sensitivities significantly reduced their affective reactivity to normally-triggering auditory and visual stimuli. Taken together, this pair of considerations suggests not only a potential common underlying neuro-affective mechanism contributing to both misophonia and MKS [see also 11, 12], but that those with MKS may show altered (e.g., heightened) responsivity to affectively-valenced visual-social stimuli.

To investigate this possibility our study adopted a classic emotional oddball task, wherein participants are asked to view a serial stream of faces shown on a computer screen that includes both frequent "standard" faces with neutral expressions and more rare or infrequent "oddball" faces having either a happy or angry expression [13]. Specifically, as reviewed by Schlüter et al. [14], in prior studies using event-related potential (ERP) measures in emotional oddball tasks, the amplitude of the P300 ERP component elicited by the "oddball" (or emotionally-expressive) faces is taken to positively scale with the magnitude of the implicit emotional reactivity to the face; the higher the P300 amplitude, the greater the indexed emotional reaction. Further, this P300 response to "oddball" emotional faces shows a sensitivity to individual variability in factors such as personality traits [15], and subclinical levels of alexithymia and depression [16]. Given these established findings, we thus reasoned that not only would the P300 amplitude elicited by "oddball" faces in the context of an emotional oddball task be a valid measure or operational definition of emotional responsivity to affectively-valenced visual-social stimuli, but that it also had the potential to capture possible impacts of individual variability in MKS on that affective responsivity.

We thus asked participants to perform an emotional oddball task while recording their ERPs to the faces. If MKS is indeed associated with a heightened affective reactivity to visual-social stimuli, it predicted that those participants reporting higher levels of MKS should show a larger P300 amplitude in their response to the emotionally-deviant "oddball" faces, relative to those participants reporting lower or no levels of MKS.

## Methods

### Participants

Participants were recruited between February and June of 2022 through the community via posting at the Paid Participants Study List hosted by Psychology Graduate Student Council website, and remunerated $20 (CAD) for their participation. Written informed consent was obtained from all participants included in the study. An *a priori* power analysis was conducted [G*Power; 17] based on the auditory ERP oddball experiment in misophonics by Schröder [18] with a medium effect size of (0.41) at a significance level of 0.05. The power analysis determined that a sample size of 38 participants would be required to achieve a power of 0.90. We thus recruited a total of 46 participants, of which 7 were excluded due to excessive eye movement and other recording artifacts identified during initial data analysis. The remaining 39 participants (24 female, 1 gender non-conforming, 1 non-binary), had no history of neurological problems, and had normal or corrected-to-normal vision. UBC Behavioural Review Ethics Board approved all procedures and protocols of this experiment.

### Stimuli and task

**Fig 1:**
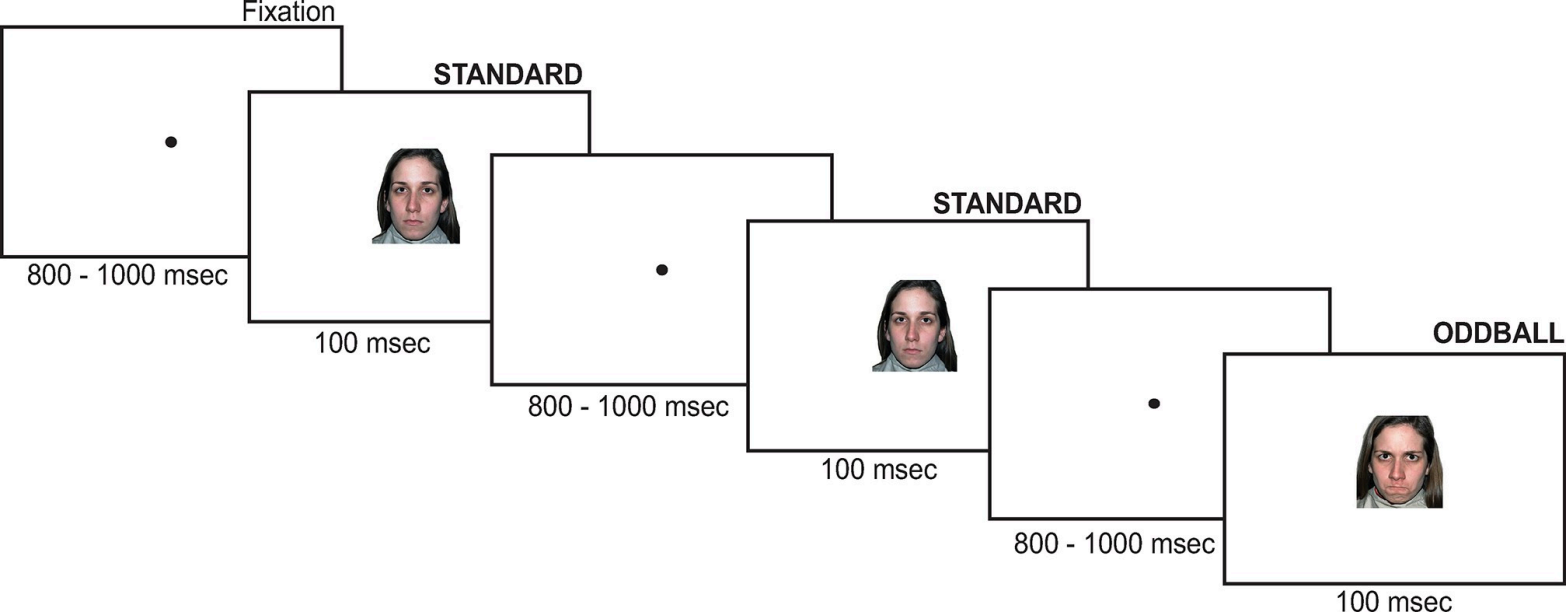
Task paradigm. Timing and sequence of rare, deviant stimulus (i.e., target, “angry face”) and common, standard (i.e., nontarget, “neutral face”) in the present emotional oddball task.

Participants performed a visual emotional oddball task adapted from Campanella and colleagues [19] in which participants observed a frequent, standard stimulus (a neutral face) and three infrequent, oddball stimuli that were responded to with a button press. The oddball stimuli changed either on emotion (same identity, happy or angry expression), or on identity (different identity, neutral expression). The four faces (two females) with neutral, happy, and angry expressions were selected from the highly standardized set of pictures from the NimStim Set of Facial Expressions [20]. The task involved presentation of a serial stream of stimuli at foveal centered fixation dot, against a white background. Participants were asked to make a manual button press with their right thumb for oddball stimuli and asked to withhold a button press response when presented with standard stimulus. The timing and sequence of stimuli for the task are shown in Fig 1. Each participant completed 5 blocks of 100 trials, in which the standard (neutral face) appeared 82 times, and each of the oddball appeared 6 times (i.e., 6 * happy face, 6 * angry face, 6 * different identity face). The stimulus displayed for 100 msec, and the inter-stimulus interval was jittered on a trial-by-trial basis between 800 and 1000 msec for an average ISI of 900 msec.

### Procedure

Participants provided written consent, then got prepared for recording his or her electroencephalogram (EEG), and instructed in the oddball task while seated at the task computer running MATLAB R2010a [21] with Psychtoolbox [22–24]. Once commenced, the oddball task took approximately 1 hour, giving participants a total study time of approximately 2 hours which included the setup, task, and clean up for the participant. The participants were measured to be seated 57 cm from the centre of the computer screen, and asked to press a button on the joystick using their right thumb for the oddball stimuli, and withhold button press for standard stimulus. In addition to recording the P300 ERP waveforms generated by oddballs, we also assessed behavioural responses such as reaction times and accuracy rates. Following the completion of the oddball task participants filled out misokinesia assessment questionnaire (MkAQ), a 21-item instrument that calculates an individual’s level MKS [2]; scores on the MkAQ were then used to identify our low and high MKS groups for data analyses as reported below.

### Electrophysiological recording and analysis

Continuous EEG was recorded during the task via 64 Ag/AgCl active electrodes mounted in an elastic cap (BioSemi Active-Two amplifier system; BioSemi, Amsterdam, Netherlands) in spatial accordance with the international 10–20 system. Two additional electrodes located over the medial-parietal cortex (Common Mode Sense and Driven Right Leg) are used as ground electrodes. Recordings are digitized at 256 Hz, digitally filtered offline between 0.1 and 30 Hz (zero phase-shift Butterworth filter) and then referenced offline to the average of two mastoid electrodes. EEG data processing is performed using ERPLAB [25], a toolbox within MATLAB 2014a [26] used in conjunction with EEGLAB. To ensure proper eye fixation and allow for the removal of events associated with eye movement artifacts, horizontal electrooculograms (EOGs) are also recorded—the horizontal EOGs from two electrodes on the right and left outer canthus. As participants were wearing masks for the duration of our study, vertical EOG was not recorded as it is typically recorded from an electrode inferior to the right eye. Offline, computerized artifact rejection was used to eliminate trials during which detectable eye movements and blinks occurred. Eye movements or muscle artifacts were automatically rejected from analysis, using the moving windows peak-to-peak option in ERPLAB, with amplitude thresholds customized for each participant (range 100–250 μV). Statistical analyses of the ERP waveforms reported below focused *a priori* on the P300 ERP component, based on mean amplitude measures using a 500 to 600 ms post-stimulus time window, which centered on the approximate peak of the P300 as identified in the grand-averaged waveforms; all measures were relative to a -200 to 0 pre-stimulus baseline.

## Results

### MKS grouping

**Fig 2:**
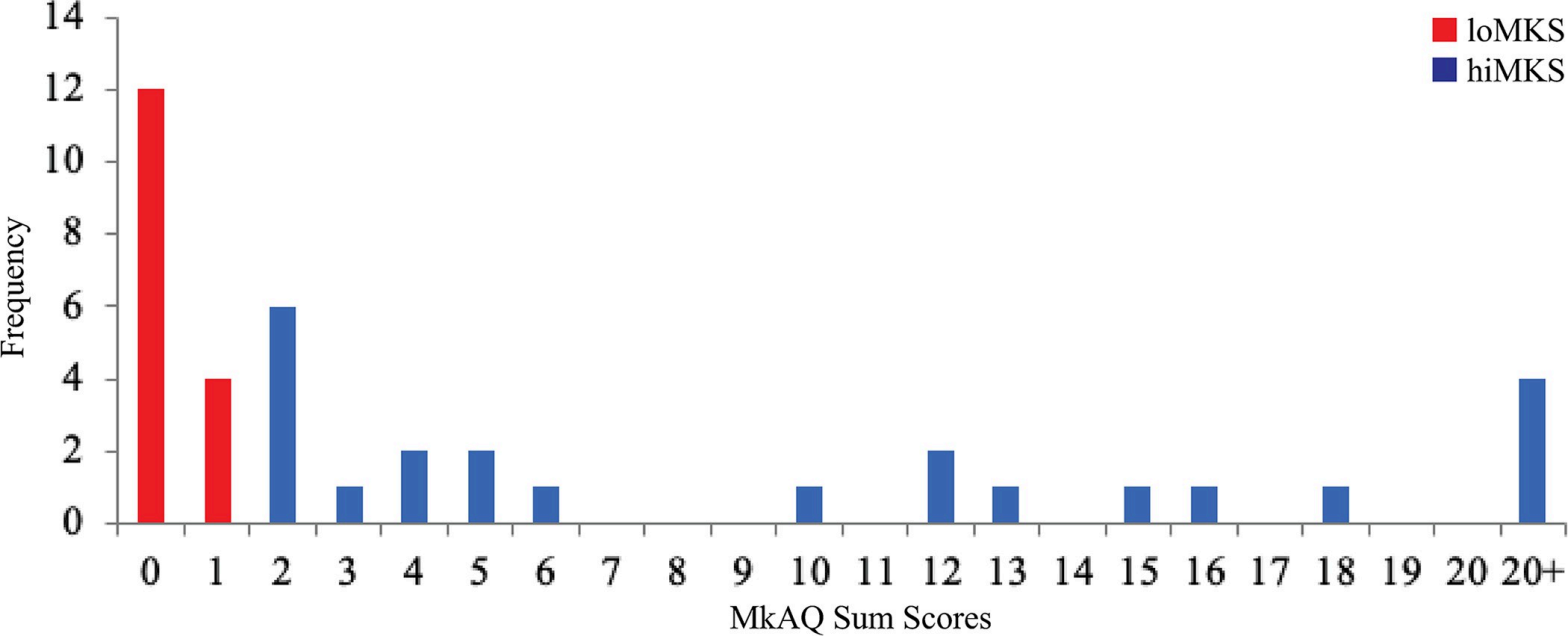
Frequency of MkAQ sum scores.

Adopting the same grouping protocol as in our previous study of MKS [2], we summed the participants scores on the MkAQ (Fig 2) and then split the participants into two groups, where participants having sum scores of 0–2 were labeled the “low misokinesia” group (loMKS; 15 women, 1 gender non-conforming; age, mean = 23.32, SD = 8.46) and those with sum scores of 3+ were labeled “high misokinesia" group (hiMKS; 9 women, 1 non-binary; age, mean = 22.06, SD = 3.58).

### Behaviour

Reaction times to the oddball faces, hit rates for the oddball faces, and false alarm rates to the standard faces (or faces not requiring a response) are reported in Table 1 as a function of group and face condition. For reaction times, a repeated-measures ANOVA with MKS group (loMKS vs. hiMKS) included as a between-groups factor, and oddball type (angry faces, happy faces, and different identity faces) included as within-groups factors found a main effect of oddball type (*F*(1,37) = 5.760, *p* = 0.022, $\eta_{p}^{2}$ = 0.135), but no main effect of group (*F*(1,37) = 0.006, *p* = 0.939) or group x oddball type interaction (*F*(1,37) = 2.1, *p* = 0.156). For hit rates to the oddball faces we conducted a similar repeated-measures ANOVA, and again found a significant main effect of faces (*F*(1,37) = 4.33, *p* = 0.044, $\eta_{p}^{2}$ = 0.105), but no main effect of group (*F*(1,37) = 0.013, *p* = 0.909) or group x oddball type interaction (*F*(1,37) = 3.468, *p* = 0.071). Finally, for false alarm rates to the standard faces, we conducted an independent samples t-tests comparing between the low and high MKS groups, but found no significant between-group difference in this accuracy measure either [*t*(37) = -0.664, *p* = 0.511].

**Table 1:** Behavioural results.

		MKS group	
	Face Condition	loMKS	hiMKS
Reaction Time (ms)	Happy Face	374 (210)	373 (204)
	Angry Face	360 (203)	333 (204)
	Different Identity Face	373 (207)	377 (205)
Hit Rate	Happy Face	0.66 (0.370)	0.651 (0.350)
	Angry Face	0.641 (0.361)	0.582 (0.348)
	Different Identity Face	0.653 (0.364)	0.661 (0.355)
False Alarm Rate	Neutral Face	0.004 (0.005)	0.003 (0.004)

Mean and standard deviations (in parentheses) of reaction time (in milliseconds) to the oddball faces, hit rates for the oddball faces, and false alarm rates for standard stimulus, presented as a function of MKS group (loMKS vs. hiMKS).

### Electrophysiology

**Fig 3:**
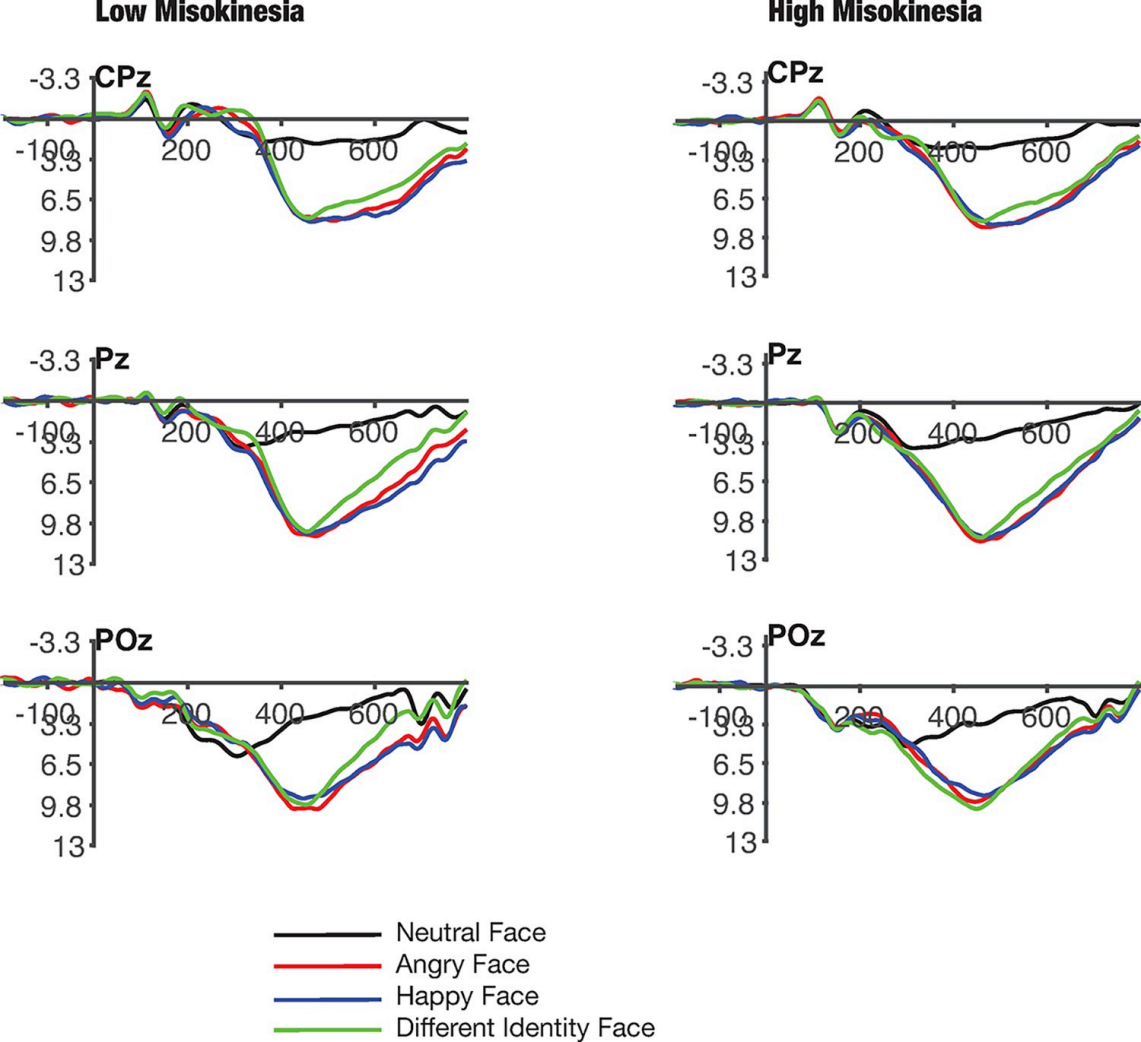
ERP waveforms for low and high misokinesia groups. Averaged waveforms showing cognitive effects of P300 ERP components elicited by targets at 3 electrode sites (CPz, Pz, and POz) as a function of MKS group (loMKS vs. hiMKS), and stimuli (neutral, angry, happy, and different identity face).

The ERP waveforms elicited by faces are shown in Fig 3 as a function of group and face type at midline central/parietal scalp electrode sites CPz, Pz, POz where the P300 ERP component is typically maximal [27, 28], and mean amplitude of the P300 ERP component within each group and condition at these electrode sites are reported in Table 2. It appeared that while both groups showed the expected P300 responses to the three oddball stimuli (angry faces, happy faces, and different identity faces), there appeared to be no salient differences in these responses between groups. This data pattern was confirmed in an omnibus repeated-measures ANOVA with MKS group (loMKS vs. hiMKS) as a between-groups factor, and face type/condition (neutral, angry, happy, and different identity) and electrode site (CPz, Pz, POz) as within-groups factors. There was an overall significant main effect of faces [*F*(3, 222) = 118.275, *p* < 0.001, $\eta_{p}^{2}$ = 0.762], but there was no significant main effect of MKS group [*F*(1, 37) = 0.03, *p* = 0.864] or a significant interaction between faces and MKS group [*F*(3, 222) = 0.089, *p* = 0.966].

**Table 2:** ERP results.

		MKS group	
P300 Amplitude	Faces	loMKS	hiMKS
CPz	Neutral Face	1.786 (1.203)	1.525 (1.51)
	Angry Face	8.032 (3.903)	8.006 (3.107)
	Happy Face	8.138 (3.98)	7.904 (2.82)
	Different Identity Face	7.176 (3.74)	7.008 (2.265)
Poz	Neutral Face	1.707 (1.753)	1.833 (2.056)
	Angry Face	7.537 (4.057)	7.781 (5.42)
	Happy Face	7.698 (4.575)	8.207 (4.496)
	Different Identity Face	6.609 (3.48)	7.3 (3.278)
Pz	Neutral Face	2.055 (1.332)	1.888 (1.63)
	Angry Face	9.296 (4.031)	9.612 (4.331)
	Happy Face	9.679 (4.028)	9.92 (3.632)
	Different Identity Face	8.102 (3.353)	8.542 (2.967)

The mean P300 amplitudes (and standard deviation) for targets as a function of MKS group (loMKS vs. hiMKS), electrodes (CPz, Pz, POz), and stimuli (neutral, angry, happy, and different identity face). Mean amplitudes were taken across a 500–600 ms post-stimulus time window, measured relative to a -200 to 0 baseline.

Given this initial null between-group finding, we did a series of follow-up analyses to confirm that this was a valid null result. First, in order to ensure that our initial findings were not an artifact of the selection of our time window for measuring mean P300 amplitude, we repeated our initial omnibus ANOVA using a 400-500ms post-stimulus measurement window. Once again there was an overall significant main effect of faces [*F*(3, 222) = 123.58, *p* < 0.001, $\eta_{p}^{2}$ = 0.770], but there was no significant main effect of MKS group [*F*(1, 37) = 0.08, *p* = 0.778] or a significant interaction between faces and MKS group [*F*(3, 222) = 0.281, *p* = 0.839].

Second, in order to control for individual and group differences in overall magnitude of the P300 responses, and to more closely examine possible group differences in responses to the oddball faces, we computed difference waveforms that subtracted the mean amplitude of the P300 for the standard/neutral face from the mean amplitude of the P300 for each oddball face [i.e., angry, happy, or different identity face; see 29] as measured across our original 500ms-600ms post-stimulus time windows. We then conducted a third mixed- model, repeated-measures ANOVA on these difference scores. Although we did find an overall significant main effect of faces [F(2, 148) = 8.00, p < 0.001, $\eta_{p}^{2}$ = 0.178], indicating that there was variability in the magnitude of the P300 responses to the different oddball faces, there was no significant main effect of MKS group [F(1, 37) = 0.116, p = 0.735] or a significant interaction between faces and MKS group [F(2, 148) = 0.039, p = 0.96].

Finally, to illuminate the possibility that our null between-groups results was due to low power in our between group analyses, we ran a series of correlations on the MkAQ sum scores with the P300 mean amplitude difference score for each of the oddball (angry, happy, different identity face) stimuli at the three electrode sites (CPz, Pz, POz) at the 500ms– 600ms time window. Across all 9 correlations (3 face types x 3 electrode sites), none showed a significant correlation (*r* ≤.095, *p*≥0.565).

## Discussion

Our study aimed to investigate whether individuals with misokinesia sensitivity (MKS) are more prone to having their attention drawn by affectively-valenced visuo-social stimuli. Participants performed an emotional oddball task that included faces with either angry or happy expressions as the deviant stimuli, while we recorded their brain electrical responses via ERPs. We found that the amplitude of the P300 ERP component elicited by these deviant "oddball" faces did not vary with the level of the participants’ self-reported MKS. The current study, consistent with our prior investigation [2] indicates a lack of attentional orienting variability associated with MKS. Moreover, we could not ascribe the present null finding to insufficient statistical power, the choice of time-window for ERP analysis, or the between-group ANOVA used for data analysis. Rather, our data suggest that MKS is not associated with heightened orienting reactions to unexpected emotionally-salient events. Given our conclusion, several critical points follow.

First, while our findings suggest MKS is not associated with a heightened sensitivity to social-affective stimuli, we do not want to over-generalize from this result in terms of what to conclude regarding emotional reactivity in MKS. For example, if MKS is indeed associated with negative emotional reactions to "triggering" stimuli as responses in the MkAQ suggest, these subjective reactions may be qualitatively distinct from the more automatic or implicit orienting reactions to emotionally-salient events that were assessed via the P300 ERP response in our study. Consistent with this possibility, the experience of misophonia has been associated with anger and disgust reactions to "triggering" events [30; see also 31, 32], subjective emotional experiences that do not likely translate into what is operationally captured in the emotional oddball paradigm. Likewise, the visual stimuli used in our study––static, non-moving faces––do not necessarily align with the typical kinds of kinetic "triggering" stimuli like fidgeting that are associated with MKS [2]. In other words, our study was not assessing orienting responses to those kinds of stimuli that engender emotional reactions in those with MKS. So again, while our data suggest MKS is not linked to a heightened orienting reactivity to emotionally-salient visual-social stimuli in general, this does not preclude either heightened orienting reactions to "triggering" types of stimuli, or emotionality in the subjective experience of MKS.

Second, and relatedly, in assessing orienting reactions to emotionally-salient visual events in the context of the emotional oddball task, our study was specifically designed to examine a potential mechanistic contributor to MKS. In finding no evidence to support this possibility, the current results parallel our prior finding that MKS is also not associated with another possible underlying visual orienting mechanism––reflexive visuo-spatial orienting to sudden-onset visual events [2]. The absence of an association between MKS and reflexive visuo-spatial orienting to sudden-onset visual events, combined with the null findings in our present study, suggests that MKS may be associated with more selective stimulus reactions, and ones that may vary across individuals, in that different people report having significantly different triggers for their MKS [33]. As such, MKS may not simply reflect population-level differences in how strongly those with vs. without MKS orient to a narrow class of visual events, such as faces.

Another potential mechanistic possibility is that MKS may be associated not with the strength of orienting to visual events, but conversely, to difficulties in disengaging attention from triggering stimuli. In particular, clinically-oriented research has consistently shown that individual with anxiety-related challenges have specific deficits in their ability to disengage not just their thoughts from negative or threatening information [34, 35], but their visual attention [36]. In the realm of visual attention, our findings align with the separation of orienting mechanisms, as explored in this study, from mechanisms related to attentional disengagement. For instance, an individual with misokinesia might orient their attention to a fidgeting stimulus in a normative manner, but perhaps they may experience difficulty in disengaging from the stimulus once attention has been captured.
