# Supplementary material for: Is misokinesia sensitivity explained by visual attentional orienting? ERP evidence from an emotional oddball task suggests no
Source: PLoS One. 2024 Jul 29;19(7):e0306464. doi: 10.1371/journal.pone.0306464 (PMC11285937; doi:10.1371/journal.pone.0306464)
Supplement: Attachment — Submitted filename: Response to Reviewers.docx [file pone.0306464.s001.docx]

*When completing the data availability statement of the submission form, you indicated that you will make your data available on acceptance. We strongly recommend all authors decide on a data sharing plan before acceptance, as the process can be lengthy and hold up publication timelines. Please note that, though access restrictions are acceptable now, your entire data will need to be made freely accessible if your manuscript is accepted for publication. This policy applies to all data except where public deposition would breach compliance with the protocol approved by your research ethics board. If you are unable to adhere to our open data policy, please kindly revise your statement to explain your reasoning and we will seek the editor&#39;s input on an exemption. Please be assured that, once you have provided your new statement, the assessment of your exemption will not hold up the peer review process.*

The data and materials necessary to reproduce the findings reported in this manuscript are available at https://osf.io/8j7mf/.

**A Shared Reviewer Concern**

Reviewer 1: *More description and discussion should be added to illustrate the motivation of current work.*

Reviewer 2: *The introduction does not adequately acknowledge or discuss relevant prior studies that have explored the performance of Misokinesia people on attentional sensitivity (within or without in an emotional oddball task), or P300 ERP component. I recommend that the authors include a more thorough literature review to acknowledge and contextualize these prior studies. This would help situate the current research within the existing body of knowledge and provide a clearer rationale for why the study focuses on attentional sensitivity of emotional stimuli and the use of P300 ERP component.*

We appreciate this shared reviewer concern here. However, as we have now tried to better emphasize in our revision, we now stress in the opening paragraph that quite literally our study is only the second-ever (to our knowledge) experimental investigation specifically addressing possible mechanistic contributors to misokinesia, and so there is literally nothing for us to expand on by way of description or discussion for our work's motivation with respect to misokinesia -- we know virtually nothing at this point, and so are doing initial preliminary studies. We now state in our opening paragraph:

*Although approximately one-third of the general North American population appears to self-report at least some degree of misokinesia sensitivity (MKS), our recent reporting of this prevalence rate and data suggesting that misokinesia is not associated with altered attentional responses to sudden-onset visual events (2) remains the only published work to date specifically addressing the potential mechanistic basis for MSK. As such, we have yet to establish the cognitive and/or affective factors that may help to explain this widespread visuo-social sensitivity and inform intervention strategies.*

However, we also stress here and in our paper that our work was motivated and informed by two key known points about the affective contributors/correlates to misophonia sensitivity, the auditory analog to misokinesia, which we discuss and outline in our Introduction as the primary logic for our investigation of affective reactivity in misokinesia:

*Given the literal absence of empirical work on MSK, our study was informed by two lines of reasoning drawn from the literature on misophonia, misokinesia's auditory counterpart which has received increasing research interest over the past decade (1). First, although the strong negatively-valenced affective responses to auditory triggers in misophonia may modulate with the identity of the individual making the sound (e.g., a family member vs. a stranger 3–6) heightened affective reactivity itself is a core defining feature of the misophonic response (7–9). Second, in a recent case study, Webb (10) reported that administering the* $\beta$*-blocker Propranol to an individual with severe misophonic and misokinesic sensitivities significantly reduced their affective reactivity to normally-triggering auditory and visual stimuli. Taken together, this pair of considerations suggests not only a potential common underlying neuro-affective mechanism contributing to both misophonia and MKS (see also 11, Webb & Keane, 2022), but that those with MKS may show altered (e.g., heightened) responsivity to affectively-valenced visual-social stimuli.*

**Reviewer #1's Other Concerns:**

*2. Some description about the experimental design in section Introduction should be moved to section Method and materials.*

We thank the reviewer for pointing this out, and have removed unnecessary methodological details from our revised Introduction.

*3. In the section Introduction, the authors mentioned that the visuo-social sensitivity of misokinesia may be caused by cognitive-affective factors, which is actually the kernel arguments they want to prove in current study. Thus, I think the authors should elaborate on its derivation and outcomes and make this work more convincible.*

Once again, we note in the above cited paragraph from our Introduction, we explain the derivation for our study as it relates to what is known about the affective contributors to misophonia. However, we would also like to gently point out here that we are not attempting to prove anything about misokinesia sensitivity in our study. We are trying to determine whether any preliminary evidence can be found regarding possible differential affective responsivity in those with vs. without misokinesia sensitivity to one class of basic visual-social stimuli (emotionally expressive faces), and we believe that what we review regarding misophonia makes this simple goal reasonable and justified as a scientific exercise. Quite simply, we don't know if there are affective contributors to misokinesia sensitivity, but the small bit of work done on misophonia suggests there may be, so we designed the first-ever study to see if that might be the case.

4. *The detailed information about EEG preprocessing should be added. In fact, the waveforms of ERP signals may be influenced by various noises, which may be the reason of null ERP finding. Besides the EEG reference methods may also influence the waveform of ERP.*

With respect to the concern about providing detailed information about our EEG/ERP pre-processing, we gently note here that we did in fact include the following paragraph in our Methods section, which describes all our pre-processing steps, including the use of an average mastoid reference, which is a long-standing and highly-common (i.e., not unusual) choice for scalp electrode referencing in ERP studies:

*Continuous EEG was recorded during the task via 64 Ag/AgCl active electrodes mounted in an elastic cap (BioSemi Active-Two amplifier system; BioSemi, Amsterdam, Netherlands) in spatial accordance with the international 10–20 system. Two additional electrodes located over the medial-parietal cortex (Common Mode Sense and Driven Right Leg) are used as ground electrodes. Recordings are digitized at 256 Hz, digitally filtered offline between 0.1 and 30 Hz (zero phase-shift Butterworth filter) and then referenced offline to the average of two mastoid electrodes. EEG data processing is performed using ERPLAB, a toolbox within MATLAB 2014a (The MathWorks, Inc.) used in conjunction with EEGLAB. To ensure proper eye fixation and allow for the removal of events associated with eye movement artifacts, horizontal electrooculograms (EOGs) are also recorded—the horizontal EOGs from two electrodes on the right and left outer canthus. As participants were wearing masks for the duration of our study, vertical EOG was not recorded as it is typically recorded from an electrode inferior to the right eye. Offline, computerized artifact rejection was used to eliminate trials during which detectable eye movements and blinks occurred. Eye movements or muscle artifacts were automatically rejected from analysis, using the moving windows peak-to-peak option in ERPLAB, with amplitude thresholds customized for each participant (range 100– 250 μV).*

We also do appreciate the reviewers suggestion that residual noise in the waveforms may provide one explanation for our null finding regarding the impact of misokinesia sensitivity on P300 amplitude.  Indeed, this is a common issue in many ERP data sets.  However, visual inspection of the ERP waveforms included in our paper show virtually no residual noise in the pre-stimulus baseline portion of the waveforms, the portion of the waveform where noise levels are typically assessed (e.g., Handy, 2004; Luck, 2005).  Rather, in light of the control analyses we include to rigorously assess the validity of our null between-group finding, we believe our data remain consistent with our conclusion -- misokinesia sensitivity did not correlate or otherwise co-vary with P300 amplitude in this paradigm.

5. *I’m wondering that whether the experimental design and the selection of ERP stimulus are reasonable. I think the authors should elaborate on this.*

We appreciate that in our original submission our discussion of the emotional oddball paradigm in the Introduction focused more on methodological detail and less on explaining our rationale for selecting the emotional oddball task for our study. As such, we have now extensively revised this portion of our Introduction, which also emphasizes that this task has been widely used in the neurocognitive and neuroaffective literature, which further justifies its selection here, owing to the strong and well-established basis for how to functionally interpret the primary dependent variable in the paradigm -- the amplitude of the P300 ERP component elicited by the faces:

*To investigate this possibility our study adopted a classic emotional oddball task, wherein participants are asked to view a serial stream of faces shown on a computer screen that includes both frequent "standard" faces with neutral expressions and more rare or infrequent "oddball" faces having either a happy or angry expression (12). Specifically, as reviewed by Schlüter et al. (15), in prior studies using event-related potential (ERP) measures in emotional oddball tasks, the amplitude of the P300 ERP component elicited by the "oddball" (or emotionally-expressive) faces is taken to positively scale with the magnitude of the implicit emotional reactivity to the face; the higher the P300 amplitude, the greater the indexed emotional reaction. Further, this P300 response to "oddball" emotional faces shows a sensitivity to individual variability in factors such as personality traits (16) and subclinical levels of alexithymia and depression (17). Given these established findings, we thus reasoned that not only would the P300 amplitude elicited by "oddball" faces in the context of an emotional oddball task be a valid measure or operational definition of emotional responsivity to affectively-valenced visual-social stimuli, but that it also had the potential to capture possible impacts of individual variability in MKS on that affective responsivity.*

**Reviewer #2's Other Concerns:**

*2. How the 39 participants are divided into two groups, low misokinesia group and high misokinesia group? What is the screening criterion? Although the authors introduce the misokinesia assessment questionnaire at the paragraph of Electrophysiological in Results, it is more appropriate to introduce in advance at the paragraph of Participants in Methods. Details about the sum scores of each group also need report. I recommend that the authors include a table or graph to describe the individual level on the misokinesia assessment questionnaire.*

We thank the reviewer for pointing this out, and we have added the following paragraph in the methods section. Additionally, we have included a subsection in the results on the MKS groupings where we explain how the participants were divided into the two groups, and well as a graph that describes the individual level scores on our questionnaire.

*Following the completion of the oddball task participants filled out misokinesia assessment questionnaire (MkAQ), a 21-item instrument that calculates an individual’s level MKS (2); scores on the MkAQ were then used to identify our low and high MKS groups for data analyses as reported below.*

# *Results*

## *MKS Grouping*

*Adopting the same grouping protocol as in our previous study of MKS (2), we summed the participants scores on the MkAQ (see Figure 2) and then split the participants into two groups, where participants having sum scores of 0-2 were labeled the “low misokinesia” group (loMKS; 15 women, 1 gender non-conforming; age, mean = 23.32, SD = 8.46) and those with sum scores of 3+ were labeled “high misokinesia" group (hiMKS; 9 women, 1 non-binary; age, mean = 22.06, SD = 3.58).*

*Figure 2. Frequency of MkAQ sum scores.*

*3. Is there a significant difference among standard neutral face, happy face, angry face and different identity face condition in reaction time and accuracy? The authors report the P300 mean amplitude under the four conditions, why they missed the comparison on behavior data.*

We thank the reviewer for pointing out the need for better inclusion of the behavioural data. We now include the reaction times to oddball faces, the hit rate for oddball faces, and false alarm rate to standard faces in Table 1.

***Table 1. Behavioural results***

*Mean and standard deviations (in parentheses) of reaction time (in milliseconds) to the oddball faces, hit rates for the oddball faces, and false alarm rates for standard stimulus, presented as a function of MKS group (loMKS vs. hiMKS).*

*4. What kind of statistic analysis has been used to the behavioral data? Note it in an appropriate place.*

We thank the author for this suggestion and we have amended our results section to show the specific statistical analyses we used in the behavioural data.

*Reaction times to the oddball faces, hit rates for the oddball faces, and false alarm rates to the standard faces (or faces not requiring a response) are reported in Table 1 as a function of group and face condition. For reaction times, a repeated-measures ANOVA with MKS group (loMKS vs. hiMKS) included as a between-groups factor, and oddball type (angry faces, happy faces, and different identity faces) included as within-groups factors found a main effect of oddball type (F(1,37) = 5.760, p =0.022,* $\eta_{p}^{2}$ *= 0.135), but no main effect of group (F(1,37) = 0.006, p =0.939) or group x oddball type interaction (F(1,37) = 2.1, p =0.156). For hit rates to the oddball faces we conducted a similar repeated-measures ANOVA, and again found a significant main effect of faces (F(1,37) = 4.33, p =0.044,* $\eta_{p}^{2}$ *= 0.105), but no main effect of group (F(1,37) = 0.013, p =0.909) or group x oddball type interaction (F(1,37) = 3.468, p =0.071). Finally, for false alarm rates to the standard faces, we conducted an independent samples t-tests comparing between the low and high MKS groups, but found no significant between-group difference in this accuracy measure either [t(37) = -0.664, p = 0.511)].*

*5. Has the protocol of the visual emotional oddball task been examined on normal healthy people? In order to draw a more comprehensive conclusion that individuals with misokinesia do not have a high attentional sensitivity to affectively-valenced visual stimuli, evidence and results of standard neutral face, happy face, angry face and different identity face condition, on both the behavior performances and ERP levels from normal healthy people, also need to be discussed.*

We thank the author for the suggestion of including information regarding the visual emotional oddball being examined on healthy participants. We have now included this in our introduction.

*Specifically, as reviewed by Schlüter et al. (15), in prior studies using event-related potential (ERP) measures in emotional oddball tasks, the amplitude of the P300 ERP component elicited by the "oddball" (or emotionally-expressive) faces is taken to positively scale with the magnitude of the implicit emotional reactivity to the face; the higher the P300 amplitude, the greater the indexed emotional reaction. Further, this P300 response to "oddball" emotional faces shows a sensitivity to individual variability in factors such as personality traits (16) and subclinical levels of alexithymia and depression (17). Given these established findings, we thus reasoned that not only would the P300 amplitude elicited by "oddball" faces in the context of an emotional oddball task be a valid measure or operational definition of emotional responsivity to affectively-valenced visual-social stimuli, but that it also had the potential to capture possible impacts of individual variability in MKS on that affective responsivity.*
